# Current Status and Perspectives of Antibacterial Agents Belonging to 2-Oxazolidinones

**DOI:** 10.3390/ph19030432

**Published:** 2026-03-06

**Authors:** Jessica Ceramella, Annaluisa Mariconda, Domenico Iacopetta, Maria Marra, Alessia Catalano, Paola Checconi, Stefano Aquaro, Carmela Saturnino, Pasquale Longo, Maria Stefania Sinicropi

**Affiliations:** 1 Department of Pharmacy, Health and Nutritional Sciences, University of Calabria, 87036 Arcavacata di Rende, Italy; jessica.ceramella@unical.it (J.C.); domenico.iacopetta@unical.it (D.I.); mariamarra1997@gmail.com (M.M.); s.sinicropi@unical.it (M.S.S.); 2 Department of Basic and Applied Sciences, University of Basilicata, Via dell’Ateneo Lucano, 10, 85100 Potenza, Italy; annaluisa.mariconda@unibas.it; 3 Department of Pharmacy-Drug Sciences, University of Bari “Aldo Moro”, Via Orabona, 4, 70126 Bari, Italy; 4 Department for the Promotion of Human Sciences and Quality of Life, San Raffaele University, Via di Val Cannuta 247, 00166 Rome, Italy; paola.checconi@uniroma5.it; 5 Laboratory of Microbiology, IRCCS San Raffaele Roma, Via di Val Cannuta 247, 00166 Rome, Italy; 6 Department of Life, Health and Environmental Sciences, University of L’Aquila, Piazzale Salvatore Tommasi, 1, Blocco 11, Coppito, 67010 L’Aquila, Italy; stefano.aquaro@univaq.it; 7 Department of Health Sciences, University of Basilicata, Via dell’Ateneo Lucano, 10, 85100 Potenza, Italy; carmela.saturnino@unibas.it; 8 Department of Chemistry and Biology “A. Zambelli”, University of Salerno, Via Giovanni Paolo II, 132, 84084 Fisciano, Italy; plongo@unisa.it

**Keywords:** tuberculosis, multidrug-resistant bacteria, linezolid, tedizolid, antimicrobial resistance

## Abstract

In the last three decades, 2-oxazolidinones have emerged as an important class of inhibitors of bacterial protein synthesis, effective in the treatment of multidrug-resistant (MDR) bacterial infections. From a public health perspective, the importance of 2-oxazolidinones is related to the treatment of tuberculosis (TB), primarily MDR-TB and extensively drug-resistant XDR-TB. Linezolid, the first oxazolidinone antibiotic approved by FDA, is still used in therapy despite common adverse events, such as myelosuppression and serotonergic toxicity, as well as the increasing percentage of linezolid-resistant bacteria (*Staphylococcus aureus*, enterococci and methicillin-resistant *S. aureus*). Tedizolid phosphate was the second commercially available oxazolidinone antibiotic approved, followed by other oxazolidinones (contezolid, radezolid, ranbezolid, sutezolid, delpazolid, cadazolid, TBI-233 and MK-7762) that are in clinical study. Contezolid is approved in China and cadazolid has entered phase III clinical trials. This comprehensive review intends to provide an overview of the compounds belonging to this class already in use in therapy and/or clinical studies and to portray the most significant and recent outcomes regarding new oxazolidinones under study. Three literature databases, i.e., PubMed/MEDLINE, Google Scholar and Scopus, were used for the literature search, particularly focusing on the last five years, and screened using different keywords. The design of new drugs belonging to this class may be of considerable interest to researchers and clinicians, contributing to the discovery of new antibiotics that retain antibacterial activity but have fewer side effects.

## 1. Introduction

2-Oxazolidinones belong to the class of cyclic carbamates, a subgroup of nitrogen-containing heterocycles, which represent a key structural motif in many pharmaceuticals and agrochemicals [1]. The first studies on oxazolidinones date back to 1978, when they were studied for their efficiency in the control of plant diseases. After six years, their antibacterial activities were documented [2]. Specifically, this activity was ascertained by DuPont Pharmaceuticals at the end of the 1980s. However, the first oxazolidinones studied were not suitable for development; thus, the program was interrupted. Nevertheless, in the early 1990s, the Upjohn Corporation restarted to investigate these compounds, along with structure–activity relationship (SAR) studies and the development of analogs, specifically 2-oxazolidinones, that were not toxic but had effective antibacterial properties [3]. 2-Oxazolidinones are chiral compounds that behave as potent antibiotics against Gram-positive bacteria, acting as protein synthesis inhibitors in an initial stage that encompasses the transfer RNA (tRNA) binding process [4,5]. Specifically, these compounds bind to the 23S ribosomal ribonucleic acid (23S rRNA) on the ribosome’s 50S subunit. 2-Oxazolidinones are used in the treatment of infections caused by Gram-positive bacteria, including methicillin-resistant staphylococci and vancomycin-resistant enterococci. These compounds also demonstrated activity towards some of the most critical bacteria, specifically the Gram-positive *Enterococcus faecium* and *S. aureus*, which belong to the “ESKAPEE” group of bacteria (*E. faecium*, *S. aureus*, *Klebsiella pneumoniae*, *Acinetobacter baumannii*, *Pseudomonas aeruginosa*, *Enterobacter* species, and *Escherichia coli*), known for their ability to evade the effects of antibiotics, categorized under the acronym. However, 2-oxazolidinones are not suitable for Gram-negative pathogens, which are intrinsically resistant to these agents [6]. Currently, several 2-oxazolidinones are either in development or have already been approved for use in clinics to treat bacterial infections, including TB, which represents the thirteenth primary cause of mortality worldwide and the second most important infectious cause of death, surpassing AIDS. The importance of the 2-oxazolidinone moiety is underlined by some other compounds with diverse biological activities. Indeed, some antibacterial agents such as ketolides telithromycin, cethromycin and solithromycin [7] contain a 2-oxazolidinone ring, even though they are derivatives of macrolides; and other drugs with diverse activities, including the anticoagulant rivaroxaban [8] and the cytokine modulator cytoxazone [9] also present the 2-oxazolidinone ring. Although 2-oxazolidinones represent one of the most studied *O*-heterocycles in medicinal chemistry [10,11], they have not been given the label of “privileged drug scaffolds” yet, as they are addressed almost exclusively as antibacterials [12].

**Figure 1:**
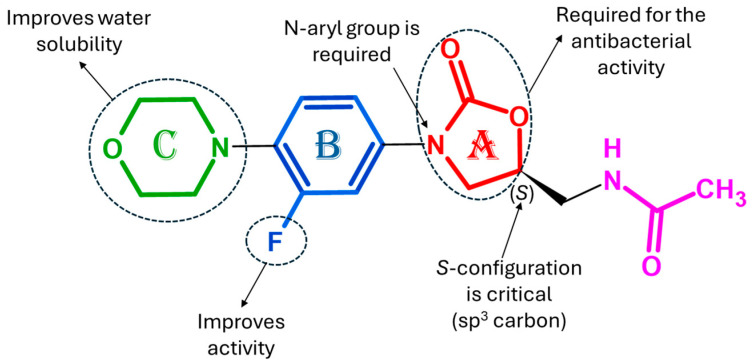
Structure of linezolid showing the important functional moieties responsible for antibacterial activity.

Linezolid (Figure 1) was the first available oxazolidinone approved by the US Food and Drug Administration (FDA) in 2000 for adults and in 2005 for pediatric use. It has now become a valuable clinical option for treating MDR Gram-positive infections, such as those caused by vancomycin-resistant *Enterococcus faecium*, vancomycin-resistant *Enterococcus faecalis*, hetero-resistant vancomycin-intermediate *S. aureus*, and penicillin-resistant pneumococcus. However, resistance to linezolid may occur in diverse bacteria, such as *S. aureus*, mycobacteria, enterococci, and less commonly *Clostridioides difficile* [13,14]. Resistance to linezolid in *S. aureus* is mediated through mutations or modifications to the bacterial target, thereby reducing the affinity of linezolid for the peptidyl transferase center (PTC) binding site or by preventing the binding of linezolid to the PTC through a ribosomal protective effect [15]. The major mechanisms of resistance to linezolid in *S. aureus* include mutations in the domain V of 23S rRNA (primarily G2576), chromosomal mutations in the *rplC*, *rplD*, and *rplV* genes (encoding the ribosomal uL3, uL4, and uL22 proteins, respectively), the exogenous acquisition of the methylase encoded by the chloramphenicol-florfenicol resistance (*cfr*) gene, the endogenous methylation or demethylation of 23S rRNA, the acquisition of *optrA* and *poxtA* resistance genes, and the existence of the LmrS multidrug efflux pump [15]. Linezolid is used for patients with bacteremia, osteomyelitis, endocarditis, and skin and soft tissue infections (SSTIs), especially when other therapies have failed [3]. Recent studies have also shown that linezolid exerts antibacterial effects on several other bacteria including *Nocardia*, *Corynebacterium*, and anaerobes, and on fungi [16]. One of the best-known uses of linezolid is the treatment of TB. It also appears to offer clinical benefits in the management of TB meningitis, particularly in critically ill patients [17]. However, linezolid resistance has been documented in both *M. tuberculosis*, the causative agent of tuberculosis, and non-tuberculous mycobacteria in many parts of the world [18]. A recent review [19] is focused on the mechanisms of linezolid resistance in mycobacteria. Most linezolid-resistant mycobacterial strains exhibit ribosomal mutations, such as *rplC*, *rrl*, and *tsnR*. Non-ribosomal mechanisms appear to be rare. It has been suggested that resistance to linezolid may be related to a mutation in the fadD32 gene, which encodes a protein that plays an essential role in mycolic acid synthesis, or to mycobacterial efflux proteins. In *C. difficile*, resistance to linezolid is conferred by the acquisition of *cfr*-like genes [20]. Intradiscal treatment has been recently suggested to deliver effective antibacterial therapy while minimizing systemic exposure in patients with chronic lower-back pain associated with Modic change type 1 [21].

There have been considerable efforts in understanding SARs of 2-oxazolidinones. However, the exact mechanism of action of linezolid is still under study [22]. Based on SAR studies that have involved modifications in the A-, B- or C-ring of linezolid, several other compounds have been studied, and newer derivatives are still under investigation. The 2-oxazolidinones resulted in positive preclinical outcomes except for posizolid and eperezolid (structure not shown) [23]. Specifically, in 2016, AstraZeneca discontinued posizolid from further development, and cancelled the planned phase 2 studies [24]. To our knowledge, no clinical studies have been performed or are being planned for eperezolid that have not progressed beyond phase 1 clinical studies [25]. Furthermore, the emergence of linezolid-resistant bacteria, primarily linezolid-resistant enterococci and linezolid-resistant *S. aureus*, has raised safety concerns due to the interference of the drug with mitochondrial protein synthesis [26]. Thus, recent studies address new linezolid analogues and oxazolidinone derivatives to increase the antibacterial spectrum and reduce side effects [27,28]. In this comprehensive review and update, we summarize 2-oxazolidinones in therapy and clinical studies, and report the most recent studies on new compounds bearing the oxazolidinone nucleus as antibacterials.

## 2. 2-Oxazolidinones in Therapy or Clinical Studies

**Figure 2:**
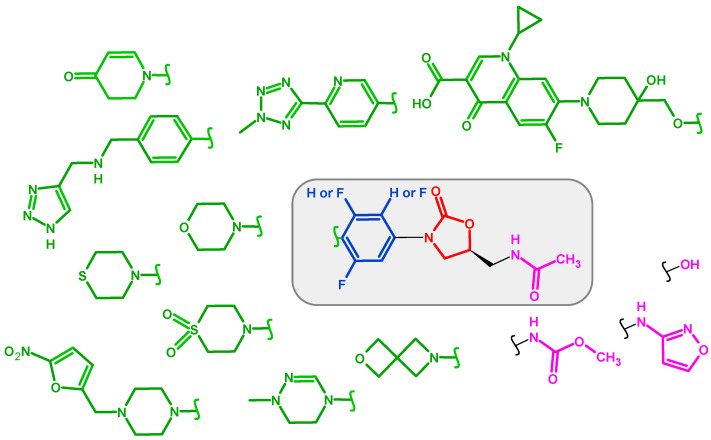
Schematic representation of the structures of 2-oxazolidinones in therapy and/or clinical trials.

**Figure 3:**
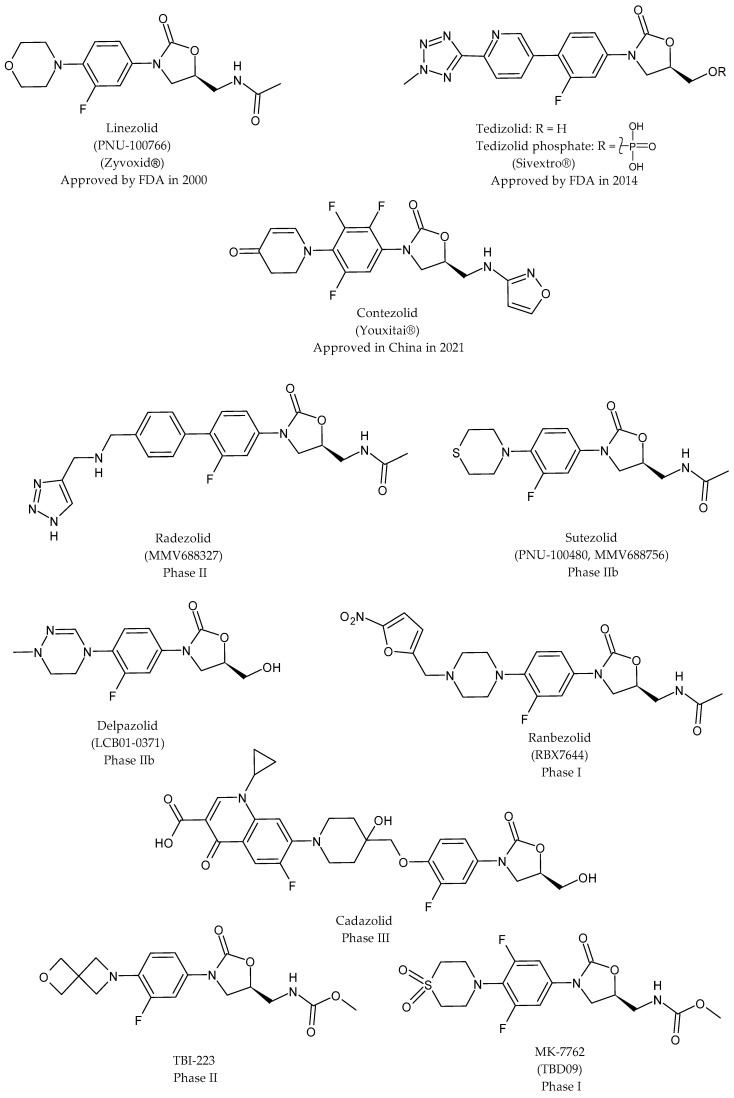
Structures of 2-oxazolidinones in therapy and/or clinical studies.

Figure 2 schematically summarizes the structures of 2-oxazolidinones in therapy and/or clinical trials, evidencing the modifications of the diverse compounds with a color-coded change at the C-ring and C-5 site. Linezolid (C_16_H_20_FN_3_O_4_, molecular weight: 337.35 g/mol, Figure 3) is considered the first member of the class of oxazolidinones. It was approved by the FDA on 18 April 2000 [29,30]. It blocks the protein synthesis of bacteria by binding to the 23S PTC of the 50S ribosomal subunit, hampering the assembly of mRNA and tRNA with the 50S and 30S subunits in the formation of the 70S initiation complex. It showed excellent in vitro activity against Gram-positive bacteria. Moreover, it was selected for further clinical development due to its good bioavailability and serum levels, which enabled twice-daily dosing, advancing to the subsequent phases of development. Linezolid underwent several structural modifications leading to reduce toxicity while preserving antibacterial activity [31,32]. The recent double-blind, randomized, placebo-controlled trial NCT05944458 suggested that intravenous N-acetylcysteine may prevent linezolid-associated thrombocytopenia, as established in 250 critically ill adults receiving linezolid for ≥48 h [33]. It has been recently recategorized by the World Health Organization (WHO) as a Group A drug, underlining its role in the treatment of MDR-TB [34] and XDR-TB, which is a severe form of TB that is resistant to the most efficacious first-line and second-line TB drugs, including isoniazid and rifampin, at least one fluoroquinolone, and an injectable second-line drug [35]. As a key component of the bedaquiline, pretomanid and linezolid (BPaL) regimen, it has shown high efficacy against MDR-TB and XDR-TB strains [36]. Nix-TB is a pivotal trial of the BPaL regimen, which received FDA approval in 2019 for the treatment of adults with highly drug-resistant pulmonary TB [37]. ZeNix is a successor of Nix-TB, which consists of a variant of BPaL with a lower dose and shorter duration than linezolid; it was evaluated if the efficacy of BPaL can be maintained while reducing toxicity. It was demonstrated to successfully cure most patients with drug-resistant TB within 6 months [38,39]. The use of linezolid as part of a BPaL and a BPaL(M) (bedaquiline, pretomanid, linezolid and moxifloxacin) regimen has been studied in several trials (NCT02333799, NCT03086486) [40,41], recommended by WHO for rifampicin-resistant/MDR-TB (RR/MDR-TB), and it is a WHO Group A drug for RR/MDR-TB [35,42].

Tedizolid phosphate (Sivextro-Cubist) is an oxazolidinone prodrug that is converted in vivo to the active form by phosphatases. It was approved on 20 June 2014 as a therapy for acute bacterial skin and skin structure infections (ABSSSIs) [43]. Tedizolid, formerly called torezolid, TR-700, or DA-7157, shows a more favorable pharmacokinetic and safety profile than linezolid [44]. It has a longer biological half-life than linezolid, requiring only one daily dose for administration [45]. Tedizolid differs from linezolid because of the presence of a pyridine ring and a tetrazole moiety. The configuration of C5 in the A-ring is (*R*) in this case, considering the different priority of the groups. The 5-R configuration on the A-ring is necessary for antibacterial activity. Its off-label use has also been reported in various infections, such as osteoarticular infections [46]. It showed interesting results in the management of nocardiosis and ventilator-associated bacterial pneumonia, acting against *Nocardia* [47]. However, tedizolid-resistant *S. aureus* and *Nocardia* species are emerging [48,49]. It demonstrated a lower incidence of bone marrow suppression and gastrointestinal adverse effects than linezolid [45]. Tedizolid has been suggested as a safer option than linezolid for managing streptococcal toxic shock syndrome due to clindamycin-resistant *Streptococcus pyogenes* in patients with comorbidities that include thrombocytopenia [50]. Although linezolid is known to reach high concentrations in the cerebrospinal fluid and is an established option for some central nervous system infections, including cerebral nocardiosis, data regarding tedizolid penetration into the cerebrospinal fluid and clinical efficacy in central nervous system nocardiosis infections remain scarce [51].

Contezolid (MRX-1-MicuRx Pharmaceuticals; the prodrug is contezolid acefosamil, MRX-4) features a dihydropyridone moiety in place of the morpholine C-ring of linezolid and two additional fluorine atoms on the B-ring, and demonstrated a markedly lower incidence of myelosuppression and MAO inhibition, while at the same time maintaining high activity against Gram-positive bacterial pathogens [52]. It has demonstrated in vitro activity against drug-sensitive and drug-resistant *M. tuberculosis* similar to that of linezolid [53]. SAR studies evidenced that the trifluorophenyl moiety and isoxazole group were essential in reducing MAO-A activity while maintaining antibacterial activity [54]. No dose modification is necessary for contezolid for patients with multidrug-resistant tuberculosis and renal insufficiency [55]. However, minimal drug–drug interactions may occur with the treatment of contezolid in complex clinical situations where multiple medications are being administered, such as tuberculous meningoencephalitis [56]. Wang et al. (2025) [57] recently reported the results of a randomized, active-controlled trial of contezolid in combination with other anti-TB drugs for treating drug-resistant TB, in comparison to linezolid. Based on the incidence of adverse events in the two-month treatment of multidrug-resistant TB, contezolid was suggested as a safer alternative to linezolid. It is currently in phase III clinical trials in the United States (NCT05369052 and NCT03747497) [58] for ABSSSIs and is approved for use in China for complicated skin and soft tissue infections (cSSTIs) [59]; it showed lower bone marrow toxicity than linezolid, a potential advantage in patients with renal failure who are prone to anemia [60,61]. Moreover, contezolid has been recently suggested as a potential anti-inflammatory agent [62].

In radezolid, the C-ring of the linezolid is replaced by a phenyl one and extended to achieve additional interaction in the PTC [63]. Wang et al. (2023) [64] reported that radezolid shows higher antibacterial and anti-biofilm activity against *S. aureus* clinical isolates from China than contezolid and linezolid. Zheng et al. (2020) [65] demonstrated that it is more active than linezolid against planktonic cells and inhibits *E. faecalis* biofilm formation [66]. Radezolid also exhibits stronger potency against *Streptococcus agalactiae* than linezolid [67].

Sutezolid (which is a thiolinezolid) [68] and delpazolid are the most recent oxazolidinones that are structurally analogues of linezolid, and demonstrate bactericidal activity and a good safety profile. They represent investigational new drugs that are being evaluated for the treatment of XDR-TB [69,70,71,72]. They are under phase IIb clinical trials in combination with bedaquiline, delamanid, and moxifloxacin for the treatment of pulmonary tuberculosis (PanACEA-SUDOCU-01 and PanACEA-DECODE-01, respectively) [73,74].

Sutezolid has completed phase 2A early bactericidal activity testing. Preliminary safety data from the SUDOCU phase II clinical trial suggest there were no dose-limiting safety issues (doses from 600 to 1200 mg daily, and from 600 to 800 mg twice daily, in combination with bedaquiline, delamanid, and moxifloxacin), and pharmacokinetic–pharmacodynamic analyses suggested there was an exposure–response relationship [73]. Sutezolid has also demonstrated activity against *Mycobacterium ulcerans* [75], the causative agent of Buruli ulcer, a neglected tropical disease affecting 33 countries worldwide [76].

Following an early bactericidal activity trial, delpazolid was evaluated in the phase 2b DECODE trial; multiple dosing schedules of delpazolid in combination with bedaquiline, delamanid and moxifloxacin were evaluated for drug-susceptible tuberculosis in 76 participants. The 1200 mg dose of delpazolid achieved the highest additive efficacy compared to the other medications. The higher dose of 800 mg twice-daily resulted in two drug-related serious side events (anemia and gastritis), both occurring in participants with relatively high delpazolid exposure [74].

Ranbezolid is a broad-spectrum antibacterial agent developed by Ranbaxy. It exerts interesting antibacterial activity against a variety of Gram-positive bacteria including methicillin-resistant *S. aureus* (MRSA), vancomycin-resistant *Enterococcus* (VRE), and *Streptococcus pneumoniae* in vitro and in vivo. It has been suggested that its cytotoxicity is caused by the conversion of nitro to nitroso radicals catalyzed by the related enzymes in vivo [77]. It has been recently demonstrated that the susceptibility of MRSA biofilm to this molecule was augmented by the co-administration with the nitroxide biofilm dispersal agent C-TEMPO (or carboxy-TEMPO, 4-carboxy-2,2,6,6-tetramethyl-1-piperidinyloxyl), thus hypothesizing that ranbezolid may work as a dual-warhead drug to eradicate biofilm, acting via the oxazolidinone mode of action as either a nitric oxide donor or cytotoxic drug thanks to its nitrofuran ring [78,79]. It has been also reported that ranbezolid, at the concentration requested for minimum biofilm eradication (16 μg/mL), acts much faster on MRSA biofilms than the combination treatment (ranbezolid with C-TEMPO), thus indicating a concentration-dependent effect on biofilm eradication, and suggesting its administration as a ‘once-daily dose’, avoiding frequent exposure to the drug [80].

Cadazolid is the first-in-class quinoxolidinone antibiotic designed for the treatment of *Clostridium difficile* infections in humans, which is one of the main causes of healthcare-associated infections worldwide [81]. It is essentially insoluble in the gastrointestinal tract, and minimal absorption of the drug through the intestinal wall has been demonstrated, leading to high drug concentrations being reached at the site of infection and avoiding potential systemic adverse effects [81]. Cadazolid exhibits a more favorable safety profile than linezolid and potent activity against *M. tuberculosis.* Its mechanisms of action and resistance parallel those of linezolid [82]. Cadazolid is in phase III clinical trials, namely NCT01987895 (IMPACT 1) and NCT01983683 (IMPACT 2) [83].

Recent literature exploring the SAR of oxazolidinones focuses on the C5 substitution of the oxazolidinone framework in order to ameliorate ribosome binding selectivity and safety profiles [84,85,86]. TBI-223 and MK-7762 (TBD09) are oxazolidinone antibiotics under clinical development for the treatment of TB. Preclinical data indicate high antituberculosis activity for TBI-223 and a possible better safety profile than linezolid [87]. It is under phase 1 clinical study (NCT03758612) in combination with bedaquiline and pretomanid. Results indicate that daily doses of 1200–2400 mg TBI-223 may achieve efficacy comparable to the BPaL regimen, with >90% of patients predicted to reach culture conversion within two months [88]. Another phase I study has been carried out to evaluate safety, tolerability and pharmacokinetic profiles in healthy subjects (NCT04865536) [23].

MK-7762 is a derivative of sutezolid, bearing a 1,1-dioxidothiomorpholine moiety and an additional fluorine on the B-ring. It shows better antitubercular activity than linezolid and limited mitochondrial protein synthesis inhibition [89]. It has been licensed from Merck & Co. by the Bill and Melinda Gates Medical Research Institute and has completed a phase I clinical trial (NCT05824091), the result of which has yet to be reported [90,91].

## 3. Structure–Activity Relationship (SAR) and Structure–Toxicity Relationship (STR) Studies of 2-Oxazolidinones

Linezolid consists of an oxazolidinone core (ring A), a 3-fluorophenyl group (ring B) linked with nitrogen, a morpholine moiety (ring C), and a methyl acetamide C-5 side chain (Figure 1). For the antibacterial activity, the N-aryl group (A + B) is required; the stereogenic center (C-5) must be in the (*S*) configuration (as depicted in the figure), and the acylamino methyl group is crucial for C-5. Moreover, the position of the fluorine atom in the B-ring is essential to enhance antibacterial activity, whereas the morpholine ring improves the pharmacokinetic profile and water solubility. SAR studies focusing primarily on the C5 side chain and C-ring of linezolid scaffold led to the discovery of tedizolid, used as the phosphate salt, contezolid, and other oxazolidinones antibacterial agents such as radezolid, ranbezolid, sutezolid, delpazolid, cadazolid, TBI-233 and MK-7762, which are currently in clinical trials. Matsingos et al. (2021) [92] evaluated the effect of various substituents on the C-5 acylaminomethyl moiety of linezolid, particularly the effect of substitution of the terminal alkyl group with aromatic, heteroaromatic and aliphatic moieties on activity, suggesting that lipophilic and smaller substituents are tolerated at this position compared to polar and larger substituents. Modifications to the C-ring and the C-5 position significantly influence both MAO inhibition and mitochondrial MPS inhibition, depending on the incorporation of suitable substituents. Several papers are focused on SARs on 2-oxazolidinones [4,10,11,12,27]. Girase et al. (2024) [86] studied SARs and STRs regarding antimycobacterial activity and serotonergic toxicity of linezolid. The presence of a two-carbon aliphatic side chain at the C-5 position was optimal, given that any increase or decrease in the chain length hindered both the activity and toxicity. Heterocyclic substitution at the C-5 position was more effective than aromatic substitution to obtain higher antimycobacterial activity and lower serotonergic toxicity. Additionally, carbonyl-substituted compounds at the C-5 position significantly reduced MAO-B inhibition compared to that of sulfonyl-substituted compounds. STR to mitigate myelosuppression and serotonergic toxicity of linezolid have been recently reviewed by Shaikh & Patel (2025) [54].

## 4. Clinical Trials Concerning 2-Oxazolidinones

Several clinical trials are ongoing that involve 2-oxazolidinones [93]. Other studies have been completed. They are summarized in Table 1. The full and detailed list of about 80 clinical trials with NCT numbers, titles, phases, and estimated completion dates is available in the Supplementary Material (Table S1). For completed trials, we only report those concluded within the last five years. Recent studies focused on combination therapies, including bedaquiline, delamanid and contezolid, BPaL, BPAL(M), BPaLMZ, BDLC (bedaquiline, delamanid, linezolid, and clofazimine), BDLLfxC (bedaquiline, delamanid, linezolid, levofloxacin and clofazimine), especially for the treatment of XDR-TB, MDR-TB, RR-TB, and TB meningitis.

## 5. Adverse Effects of 2-Oxazolidinones in Therapy

The main side effects of linezolid are myelosuppression and serotonergic toxicity. Specifically, myelosuppression derives from the inhibition of mitochondrial protein synthesis (MPS) because of the homology between bacterial and human mitochondrial ribosomes, whereas serotonergic toxicity is due to monoamine oxidase (MAO) inhibition. It is recommended that duration of treatment should be limited to a maximum of 28 days in order to prevent linezolid-related adverse effects. Thrombocytopenia is an adverse event that may occur rather frequently during linezolid treatment, as well as neutropenia and anemia [94]. Other adverse events include optic neuropathy, which can lead to visual impairment, peripheral neuropathy, hyponatremia, and lactic acidosis [95]. These effects are also generally reversible upon discontinuation of the drug, except for peripheral neuropathy [96]. Moreover, there is a potential risk of serotonin toxicity when linezolid is co-administered with other serotonergic agents [97] and, like other MAO inhibitors, linezolid can increase the risk of hypertensive crisis if patients consume foods with high tyramine content [98]. A recent systematic review found that numerous drugs have an impact on the pharmacokinetics of linezolid, likely via interaction with P-glycoprotein. Specifically, rifampicin, levothyroxine, venlafaxine, and phenobarbital could reduce the concentration of linezolid, whereas clarithromycin, digoxin, cyclosporine, proton pump inhibitors, and amiodarone could increase the concentration of linezolid [99]. Tedizolid is generally better tolerated than linezolid, requiring lower doses and demonstrating a better safety profile [49], especially when given for a short time [100]. It demonstrated a lower incidence of bone marrow suppression and gastrointestinal adverse effects than linezolid [50]. However, peripheral neuropathy may be observed during long-term suppressive therapy with tedizolid [101]. Literature studies evidenced that contezolid has improved safety and efficacy compared to linezolid and tedizolid [51]. Contezolid showed lower bone marrow toxicity than linezolid, which represents a potential advantage in patients with renal failure who are prone to anemia [60,61]. A recent study by Zhang et al. (2026) [102] evaluated the hematological effects of contezolid in patients with renal failure, evidencing that, compared with linezolid, contezolid was associated with a lower risk of clinically significant hemoglobin decline. The most common gastrointestinal and neurological side effects of contezolid are mild, primarily nausea, vomiting, and headache. In a retrospective analysis evaluating the safety and efficacy profile of contezolid in the treatment of Gram-positive bacterial infections in hematological patients, no cases of optic neuropathy, peripheral neuropathy, or myelosuppression attributable to contezolid were observed [103]. None of the clinical trials reported to date have shown nephrotoxicity or renal-related adverse events [104].

## 6. Epidemiology of Linezolid Resistance

To date, two surveillance programs have been established to study and monitor linezolid resistance in Gram-positive bacteria: LEADER (Linezolid Experience and Accurate Determination of Resistance) in the United States, and ZAAPS (Zyvox Annual Appraisal of Potency and Spectrum) as a global surveillance program. The LEADER program is a national surveillance initiative that began in 2004. It monitors the activity of linezolid alongside other Gram-positive agents, focusing on its efficacy against staphylococci, streptococci, and enterococci [105]. The LEADER program has provided annual updates on linezolid resistance mechanisms, including the identification of emerging mechanisms. Throughout the program, linezolid resistance in Gram-positive bacteria has evolved to include new species and mechanisms. According to the LEADER and ZAAPS programs, linezolid shows almost complete efficacy against enterococci. In fact, the rate of resistance to this antibiotic in enterococci is 0.22% for ZAAPS [106], and 0.78% for LEADER [107], with a lower incidence of linezolid resistance in *E. faecalis* than in *E. faecium*. No other linezolid-resistant enterococci have been detected by ZAAPS and LEADER. Most linezolid-resistant *E. faecalis* isolates were isolated in Europe and Asia, while linezolid-resistant enterococci isolates were mainly distributed in Europe and North America. Specifically, 46% of linezolid-resistant *E. faecalis* strains were documented in Europe, 31% in Asia, 16% in North America, 3% in South America and Oceania, whereas 64% of linezolid-resistant *E. faecium* isolates were reported in Europe, 27% in North America, 8% in Asia, 6%, and less than 1% in South America and Oceania [108]. Whole-genome sequencing has been recognized as a gold standard for identifying the underlying molecular mechanisms. Numerous linezolid-resistant enterococci isolates were shown to possess multiple linezolid resistance determinants and mutations, further complicating the treatment strategies. A global overview of whole-genome-sequencing-based studies summarizing all known mutational and non-mutational linezolid resistance mechanisms has been recently published by Peykov et al. (2025) [109], which focuses primarily on resistome analysis of clinical linezolid-resistant *E. faecalis* and linezolid-resistant enterococci isolates.

## 7. New Oxazolidinones Under Study

Several 2-oxazolidinones have been synthesized and/or studied as antibacterials [110], including azaspiro analogues of linezolid [111], oxazolidinone derivatives with nitrogen-containing fused heterocyclic moiety [112], and linezolid conjugates [113]. The most recent articles regarding antimicrobial activities of newly synthesized 2-oxazolidinones are summarized in Table 2. The minimum inhibitory concentration (MIC, which is the lowest concentration that resulted in maintenance or reduction in inoculum viability) is given. Studies were carried out against Gram-positive bacteria (*S. aureus*, *Staphylococcus epidermidis*, *Bacillus subtilis*, and *E. faecalis*), Gram-negative bacteria (*A. baumannii* and *P. aeruginosa*), and *M. tuberculosis* and other multidrug-resistant, non-tuberculous mycobacteria that are responsible for a wide spectrum of skin and soft tissue diseases, central nervous system infections, bacteremia, and ocular and other infections (*Mycobacterium abscessus* and *Mycobacterium smegmatis*). Moreover, the activity against resistant bacteria is studied, specifically against MRSA, MRSE, vancomycin intermediate *S. aureus* (VISA), VRE, linezolid-resistant *S. aureus*, and linezolid-resistant *E. faecalis*.

Ampomah-Wireko et al. (2025) [114] studied several oxazolidinones derived from ranbezolid, which contained a substituted *N*-methylglycyl C-ring moiety, and evaluated their antibacterial activities against *E. faecalis* and *S. aureus*. The most interesting compounds were naphthalene-substituted (**1**, (*S*)-*N*-((3-(3-fluoro-4-(4-(*N*-methyl-*N*-(naphthalen-2-ylmethyl)glycyl)piperazin-1-yl)phenyl)-2-oxooxazolidin-5-yl)methyl)acetamide) and thiazole (**2**, (*S*)-*N*-((3-(3-fluoro-4-(4-(*N*-methyl-*N*-(thiazol-2-ylmethyl)glycyl)piperazin-1-yl)phenyl)-2-oxooxazolidin-5-yl)methyl)acetamide) derivatives showing potent efficacy against *E. faecalis* (MICs = 2 µg/mL) compared to the reference compound (linezolid, MIC = 1 μg/mL against both bacteria), and also exhibiting good bacterial biofilm disruption capabilities, as demonstrated by using the SYTO-9 staining assay. The two compounds showed low toxicity toward mammalian sheep red blood cells (RBCs) (determined by measuring the 50% hemolysis, HC_50_) and human cervical (HeLa) cancer cell lines (determined by the Cell Counting Kit-8 (CCK-8) assay cells), good stability in body fluids, and long post-antibiotic effect (PAE). The mechanism of action was the disruption of the glutathione (GSH)/reactive oxygen species (ROS) balance, with ROS accumulation, which lead to membrane damage, nucleic acid leakage, and bacterial death. In another work [92], the same research group analyzed compound **3** ((*S*)-*N*-((3-(3-fluoro-4-(4-(*N*-methyl-*N*-(4-nitrobenzyl)glycyl)piperazin-1-yl)phenyl)-2-oxooxazolidin-5-yl)methyl)acetamide), also showing MIC = 2 µg/mL against *E. faecalis*, and whose results overlapped those obtained for compounds **1** and **2**.

Latterell et al. (2026) [116] described the oxazolidinones with the oxanthrene moiety. Compound **4** (*N*-{[(5*S*)-3-(7-formyloxanthren-2-yl)-2-oxo-1,3-oxazolidin-5-yl]methyl}acetamide) was studied for its inhibitory activity against *M. tuberculosis*, demonstrating that the introduction of an aldehyde moiety did not diminish antitubercular activity. Moreover, antimycobacterial activity was studied in a human monocyte-derived macrophage model of infection, and evidenced that it showed potent activity and low cellular toxicity. This compound was designed on the basis of the interesting results obtained for another oxazolidinone, **5** (*N*-(((*S*)-3-(dibenzo[*b*, *e*][1,4]dioxin-7-yl)-2-oxooxazolidin-5-yl)methyl)acetamide), reported for the first time in 2008 [123]. It inhibited the growth of Gram-positive bacteria (*E. faecalis*, *S. aureus)*, Gram-negative bacteria (*A. baumannii*), and *M. tuberculosis* with sub μg/mL potencies [117]. MIC values for both **4** and **5** were the same as for linezolid (MIC_90_ = 0.5–1.0 µg/mL).

Winkelhake et al. (2025) [118] studied oxazolidinones containing benzodioxine moieties acting against *M. abscessus*, a non-tuberculous bacterium that has recently arisen as responsible for a large spectrum of clinical manifestations, such as pulmonary infections that are increasingly common in cystic fibrosis patients [124]. Five novel oxazolidinones were obtained by the introduction of oxime, nitrile, amide, amidoxime, and oxime ester functional groups. Interestingly, the amidoxime derivative **6** (*N*-{3-[7-(*N*’-hydroxycarbamimidoyl)oxanthren-2-yl]-2-oxo-1,3-oxazolidin-5-yl}acetamide) showed in vitro antibacterial activity higher than linezolid (MIC = 16 μg/mL versus 64 μg/mL of linezolid).

Els et al. (2025) [28] prepared C-5 side chain modified derivatives via the microwave-assisted synthetic route, and evaluated their antibacterial activity against *M. smegmatis* PJV 53. This is used as a surrogate organism in tuberculosis studies as a simple approach to drug discovery for combating *M. tuberculosis*, given the non-pathogenic nature, faster growth rate, genetic similarity, and suitability for drug sensitivity testing of *M. smegmatis*. One of the newly synthesized compounds (specifically, one of the four diastereoisomers, **7**, (–)-3-(3-fluoro-4-morpholinophenyl)-5-(1-hydroxyethyl)oxazolidin-2-one) showed antibacterial activity comparable to that of rifampicin (MIC = 8 mg/L).

Wu et al. (2025) [119] studied a structural simplification of linezolid analyzing the antibacterial effect of a very simple small molecule bearing the oxazolidinone nucleus, 3-(benzo[*d*][1,3]dioxol-4-yl)oxazolidin-2-one (**8**, 3-(benzo[*d*][1,3]dioxol-4-yl)oxazolidin-2-one), against *P. aeruginosa*. Transcriptomic analysis and quantitative real-time PCR displayed the down-regulation of quorum-sensing-controlled genes in **8**-treated model *P. aeruginosa* strain PAO1. Several quorum sensing-controlled extracellular virulence factors, such as pyocyanin, elastase, and rhamnolipid, were inhibited by **8**, which also inhibits biofilm formation and cell motilities of *P. aeruginosa*. In vivo studies were carried out using a *Caenorhabditis elegans*-infection model, and showed that compound **8** mitigated *P. aeruginosa* pathogenicity, especially against PA14, a hypervirulent strain. Moreover, the synergistic activity with other antibacterial drugs was demonstrated, as **8** improved the susceptibility of *P. aeruginosa* clinical isolates towards polymyxin B or aztreonam treatment. The suggested mechanism synergy with polymyxin B was the down-regulation of *parR*, which results in alterations in membrane composition and down-regulation of *oprD* (porin D) and *oprG* (outer membrane protein); however, it deserves further study.

Zheng et al. (2025) [120] described a series of oxazolidinones bearing quaternary ammonium fragments as antibacterial agents against a range of pathogens, including methicillin-susceptible *S. epidermidis* (MSSE), MRSE, MRSA, and VRE. Compound **9** ((*S*)-4-(4-(5-(acetamidomethyl)-2-oxooxazolidin-3-yl)-2-fluorophenyl)-1-(3,5-dimethylbenzyl)pyridin-1-ium bromide) was the most potent compound and exhibited no cytotoxicity towards human hepatoma (HepG2), Vero, or human umbilical vein endothelial cell (HUVEC) cell lines and negligible hemolytic toxicity. In addition, compound **9** showed concentration-dependent bactericidal effects against *S. aureus* ATCC 33591. Molecular modeling studies have shown that compound **9** binds to the 23S rRNA of the 50S ribosomal subunit, occupying the same hydrophobic pocket as linezolid, with a large overlap degree of the side chain. The study of the interactions between compound **9** and surrounding ribonucleotide bases indicated that it likely interacts with the RNA nucleobases in the active pocket through hydrogen bonding interactions, π-π stacking, a salt bridge, and a halogen bond. The presence of the quaternary ammonium moiety may contribute to the potent antibacterial activity. Interestingly, compound **9** also showed weak inhibition of MAO-A and MAO-B with IC_50_ values of 17.77 and 240.1 μM, respectively, opposite to linezolid, which exhibited potent inhibitory effects on MAO-B (IC_50_ = 1.618 μM).

Wu et al. (2021) [121] studied a series of tricyclic benzo [1,3]oxazinyloxazolidinones as antibacterials against resistant bacteria, specifically MRSA, MRSE, VISA, vancomycin resistant *Enterococcus* (VRE), linezolid-resistant *E. faecalis*, and *M. tuberculosis* 13946 and *M. tuberculosis* 14862. Compound **10** (*N*-(((3*S*,3a*S*)-7-(6-cyanopyridin-3-yl)-6-fluoro-1-oxo-3,3a-dihydro-1*H*,9*H*-benzo[e]oxazolo [4,3-*b*][1,3]oxazin-3-yl)methyl)-acetamide) was the most interesting of the series. The importance of the configuration of the stereogenic centers (3*S*,3a*S*) was highlighted by the inactivity of the enantiomer (3*S*,3a*R*; MIC > 64 μg/mL). Compound **10** also showed no cytotoxicity against Vero cell lines (IC_50_ > 64 μg/mL), and low hERG K+ channel inhibition, thus precluding its low QT prolongation risk. Compound **10** determined mitochondrial protein synthesis (MPS) inhibition similar to that of linezolid; no activity was observed against MAO-A and moderate activity was seen against MAO-B. The in vivo efficacy of compound **10** in a mouse model was determined, demonstrating that it showed excellent stability against mouse and human liver microsomes, with high plasma exposure, high maximal plasma concentration, appropriate half-life, and excellent oral bioavailability after oral administration. In a subsequent study, the same research group [122] described a study on other compounds of the same series, in consideration of the limit of compound **10**, that is, its half-life of 3.76 h, which indicates a twice-daily treatment regimen. The most interesting compounds were **11** (*N*-(((3*S*,3a*S*)-7-(4-cyanophenyl)-6-fluoro-1-oxo-3,3a-dihydro-1*H*,9*H*-benzo[*e*]oxazolo [4,3-*b*][1,3]oxazin-3-yl)methyl)acetamide) and **12** (methyl (((3*S*,3a*S*)-6-fluoro-7-(6-(2-methyl-2*H*-tetrazol-5-yl)-pyridin-3-yl)-1-oxo-3,3a-dihydro-1*H*,9*H*-benzo[*e*]oxazolo [4,3-*b*]-[1,3]oxazin-3-yl)methyl)carbamate). The pharmaco-kinetic profile of **11** is superior to that of linezolid, with higher exposure and a longer half-life, thus being suitable for once-daily administration. It also significantly disrupted the MRSA biofilms. In vivo studies showed that compound **11** displayed higher efficacy than linezolid at the same dose, a better survival rate of infected mice, lower bacterial loads in kidneys, and higher white blood cell and lymphocyte levels compared to the vehicle control. A significant reduction in proinflammatory cytokines (IL-6, TNF-α, and IL-1β) and an increase in the anti-inflammatory cytokine IL-10 was observed in comparison to linezolid at the same dose. Moreover, severe inflammatory cell infiltration was improved by this compound. The conclusion of the authors is that compound **11** shares certain effects with linezolid, while concurrently manifesting a distinct mechanism characterized by cell membrane damage. Compound **11** also showed cross-resistance to linezolid-resistant MRSA, but with a frequency lower than that of linezolid.

A recent study by Girase et al. (2024) [86] analyzed several linezolid bioisosteres to overcome the serotonergic toxicity due to MAO enzyme inhibition associated with linezolid. The most interesting compound was the bioisostere **13** ((*S*)-*N*-((3-(3-fluoro-4-morpholinophenyl)-2-oxooxazolidin-5-yl)methyl)ethanesulfonamide) containing an ethane sulfonamide side chain against *M. tuberculosis* H_37_Rv cells and MDR *M. tuberculosis*. It also exhibited remarkable activity against drug-resistant *M. tuberculosis* clinical isolates. Importantly, it was about seven times less toxic than linezolid toward the MAO-A and about 64 times toward the MAO-B enzyme, signifying a substantial improvement in its drug safety profile. Thus, compound **13** exhibited remarkable reductions in serotonergic toxicity compared to linezolid.

## 8. Methodology

A comprehensive literature search was performed to identify studies specifically focusing on 2-oxazolidinones as antibacterials. The search was conducted using three major academic databases, PubMed/MEDLINE, Google Scholar and Scopus, focusing on the last five years. The search criteria considered the occurrence of the association of different key-words: “2-oxazolidinones”, “oxazolidinone antibiotics”, “2-oxalidinones derivatives”, “linezolid”, “tedizolid”, “sutezolid”, “delpazolid”, “cadazolid”, “contezolid”, “ranbezolid”, “TBI-233” and “MK-7762” in association with “protein synthesis inhibitors”, “multidrug-resistant bacteria”, “Linezolid resistance”, “antimicrobial resistance”, “antibacterials”, “antimicrobials”, “antimicrobial resistance”, “clinical trials” and “clinical studies”. The most relevant studies reporting the antibacterial activity of 2-oxazolidinones derivatives, published in English language, were selected for preparing this narrative review.

## 9. Conclusions

The continued evolution of MDR bacteria to existent antibiotic treatment regimens poses a public health threat. The WHO identified 15 priority pathogens that require prompt development of new antibiotics. Nearly 500,000 people each year are estimated to develop rifampin-resistant-TB or MDR-TB. The most commonly used antibiotics for these diseases, represented by isoniazid, rifampicin, ethambutol, pyrazinamide, moxifloxacin, and bedaquiline, are failing to overcome drug resistance that continues to be a serious threat to human health. Therefore, the discovery of new and potent antimicrobial agents less likely to develop resistance is of great clinical importance. 2-Oxazolidinones, introduced for the first time in the 1980s, represented a novelty in the class of antibiotics. Linezolid, the first-in-class oxazolidinone antibacterial agent, is active against a wide range of Gram-positive bacteria, also showing excellent clinical efficacy in treating drug-resistant Gram-positive pulmonary infections. Tedizolid phosphate was the second oxazolidinone drug approved by the FDA for treating MRSA skin infections. It is generally better tolerated than linezolid and offers several advantages in terms of dosing frequency and safety profile. Linezolid and tedizolid are clinically approved for treating MDR-TB infections. However, adverse effects related to these compounds often limit their use. They include myelosuppression and serotonergic toxicity, caused by MAO inhibition, optic neuropathy, peripheral neuropathy, hyponatremia, and lactic acidosis. The emergence of linezolid-resistant enterococci, linezolid-resistant *S. aureus*, *M. tuberculosis* and non-tuberculous *Mycobacteria*, hinders the treatment of MDR infections. Other 2-oxazolidinones, such as contezolid, radezolid, sutezolid, delpazolid, TBI-233, and MK-7762, are currently in clinical trials for diverse applications and are likely endowed with better toxicological profiles. Combination therapies including linezolid (BPaL, BPaL(M), BPaLMZ, BDLC and BDLLfxC regimens) are studied, especially against MDR-TB, XDR-TB, RR-TB, and TB meningitis. Meanwhile, research on anti-TB drugs continues, with drugs belonging to diverse classes. The recent quabodepistat (OPC-167832), ganfeborole (GSK3036656, GSK-070), and BTZ-043 are under study. The nitro-dihydro-imidazooxazole anti-MDR-TB drug JBD0131 also bears an oxazolidine ring. However, our attention was focused on the class of 2-oxazolidinones. We described recently synthesized compounds belonging to this class that are undergoing preclinical studies with the hope of obtaining new compounds with fewer side effects, but retaining antibacterial activity similar to or even higher than that of the existing ones. A table summarizing the ongoing clinical trials on these compounds is detailed in the text.

## 10. Future Perspectives

2-Oxazolidinones have the potential for future development of new compounds with lower toxicity and a broader spectrum, including Gram-positive and Gram-negative respiratory tract pathogens. The ideal 2-oxazolidinone should be endowed with antibacterial activity, and at the same time, capable of not causing resistance along with low side effects. The known ability of the microbial world to adapt to numerous synthetics suggests that resistance to future antimicrobial strategies is also likely. Thus, thorough understanding of resistance mechanisms and efficient monitoring of drug resistance are essential. Looking ahead, future studies should continue to investigate SARs and STRs of 2-oxazolidinones integrating molecular, immunological, and pharmacological perspectives to inform next-generation strategies for effective microbial infections and TB control. Among 2-oxazolidinones in clinical studies, cadazolid is a very promising compound in phase III. Current clinical studies and extensive case reports have provided preliminary evidence to support the therapeutic potential of cadazolid in the management of TB, especially as a safer alternative to linezolid. With accumulating evidence from more well-designed, prospective randomized controlled trials, it is anticipated that cadazolid will assume an increasingly prominent role in the future of TB treatment paradigms. Finally, co-crystal structures may help in designing novel molecules in future to address multidrug-resistant bacteria.

## Figures and Tables

**Table 1:** Clinical studies involving 2-oxazolidinones (detailed in Supporting Information).

Compound	Completed	Recruiting	Active, Not Recruiting	Not Yet Recruiting	Enrolling by Invitation	Terminated	Total
Linezolid	11	16	3	5	2	0	37
Tedizolid	9	2	1	0	0	0	12
Contezolid	1	3	0	0	2	0	6
Radezolid	2	0	0	0	0	0	2
Sutezolid (PNU-100480)	5	1	0	0	0	2	8
Delpazolid (LCB01-0371)	1	2	1	0	0	1	5
TBI-223	2	0	0	0	0	0	2
MK-7762 (TBD09)	0	1	0	0	0	2	1
BPaL, BPAL(M)	4	4	2	2	0	0	12
Other Combination Therapies	5	3	1	4	0	0	13

**Table 2:** In vitro antibacterial activity of newly synthesized 2-oxazolidinones.

Structure	Antibacterial Activity	Reference Drug	Ref
C-ring modified			
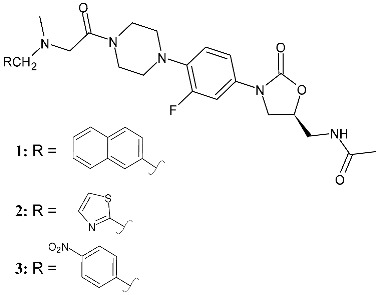	**1** ^a^ MIC = 8 µg/mL (*S. aureus*) ^a^ MIC = 2 µg/mL (*E. faecalis*)	Linezolid: ^a^ MIC = 1 µg/mL (*S. aureus*) ^a^ MIC = 1 µg/mL (*E. faecalis*)	[114]
	**2** ^a^ MIC = 8 µg/mL (*S. aureus*) ^a^ MIC = 2 µg/mL (*E. faecalis*)	Linezolid: ^a^ MIC = 1 µg/mL (*S. aureus*) ^a^ MIC = 1 µg/mL (*E. faecalis*)	[114]
	**3** ^a^ MIC = 8 µg/mL (*S. aureus*) ^a^ MIC = 2 µg/mL (*E. faecalis*)	Linezolid: ^a^ MIC = 1 µg/mL (*S. aureus*) ^a^ MIC = 1 µg/mL (*E. faecalis*)	[115]
B/C-ring modified			
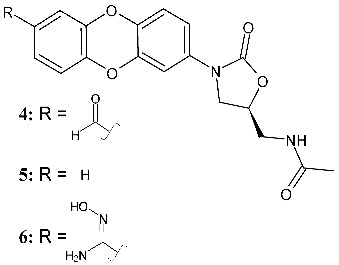	**4** ^a^ MIC_90_ = 0.5–1.0 µg/mL (*M. tuberculosis* H37Rv)	Linezolid: ^a^ MIC_90_ = 0.5–1.0 µg/mL (*M. tuberculosis* H37Rv)	[116]
	**5** ^a^ MIC_90_ = 0.25–0.50 µg/mL (*E. faecalis* ATCC 19433) ^a^ MIC_90_ = 0.5–1.0 µg/mL (*M. tuberculosis* H37Rv, 115R, 124R) ^a^ MIC_90_ = 0.25–0.50 µg/mL (*A. baumannii* 6M-1b)	Linezolid: ^a^ MIC_90_ = 1–2 µg/mL (*E. faecalis* ATCC 19433) ^a^ MIC_90_ = 0.5–1.0 µg/mL (*M. tuberculosis* H37Rv, 115R, 124R) ^a^ MIC_90_ = 0.25–0.50 µg/mL (*A. baumannii* 6M-1b)	[116,117]
	**6** ^a^ MIC = 16 µg/mL (*M. abscessus* ATCC 19977)	Linezolid: ^a^ MIC = 64 µg/mL (*M. abscessus* ATCC 19977)	[118]
C-ring and C-5 modified			
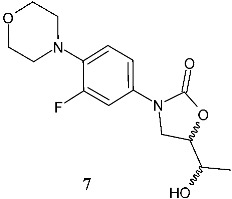	**7** ^a^ MIC = 8 µg/mL (*M. smegmatis* PJV 53)	Rifampicin: ^a^ MIC = 8 µg/mL (*M. smegmatis* PJV 53)	[28]
B/C-ring and C-5 modified			
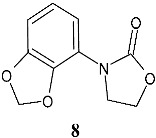	**8** At 25 µM to 200 µM concentrations, it displayed significant inhibition on the biofilm formation (30–70%) (*P. aeruginosa* PAO1)	Untreated control (*P. aeruginosa* PAO1)	[119]
C-ring modified			
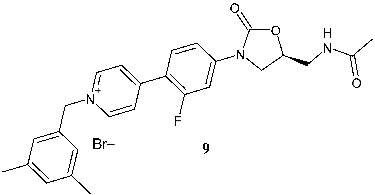	**9** ^b^ MIC = 0.12–0.25 μg/mL (MSSE) ^b^ MIC = 0.25 μg/mL (MRSE) ^b^ MIC = 4 μg/mL (MRSA) ^b^ MIC = 2 μg/mL (VRE)	Linezolid: ^b^ MIC = 1 μg/mL (MSSE) ^b^ MIC = 1 μg/mL (MRSE) ^b^ MIC = 1 μg/mL (MRSA) ^b^ MIC = 2 μg/mL (VRE)	[120]
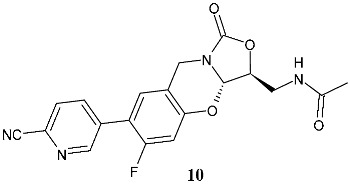	**10** ^a^ MIC = 0.25–0.5 μg/mL (MRSA) ^a^ MIC = 1 μg/mL (MRSE) ^a^ MIC = 0.25 μg/mL (VISA) ^a^ MIC = 0.25 μg/mL (VRE) ^a^ MIC = 0.48 μg/mL (*M. tuberculosis* 13946) ^a^ MIC = 0.82 μg/mL (*M. tuberculosis* 14862) ^a^ MIC = 1–2 μg/mL (linezolid-resistant *E. faecalis*)	Vancomycin: ^a^ MIC = 0.5–1 μg/mL (MRSA) ^a^ MIC < 2 μg/mL (MRSE) ^a^ MIC > 32 μg/mL (VISA) ^a^ MIC > 32 μg/mL (VRE) Isoniazide: ^a^ MIC = 2.38 μg/mL (*M. tuberculosis* 13946) ^a^ MIC > 10 μg/mL (*M. tuberculosis* 14862) Linezolid: ^a^ MIC = 4–8 μg/mL (linezolid-resistant *E. faecalis*)	[121]
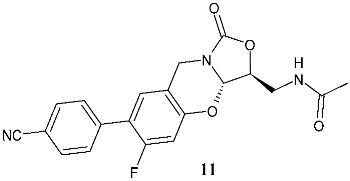	**11** ^a^ MIC = 0.5 μg/mL (*S. aureus* ATCC25923) ^a^ MIC = 1 μg/mL (*E. faecalis* ATCC29212) ^a^ MIC = 0.032 μg/mL (*B. subtilis* ATCC6633 ^a^ MIC = 0.52 μg/mL (*M. tuberculosis* H37Rv) ^a^ MIC = 8 μg/mL (linezolid-resistant *S. aureus*) ^a^ MIC = 1 μg/mL (linezolid-resistant *E. faecalis*)	Linezolid: ^a^ MIC = 2 μg/mL (*S. aureus* ATCC25923) ^a^ MIC = 0.5 μg/mL (*E. faecalis* ATCC29212) ^a^ MIC = 0.063 μg/mL (*B. subtilis* ATCC6633 ^a^ MIC = 0.8 μg/mL (*M. tuberculosis* H37Rv) ^a^ MIC = 8 μg/mL (linezolid-resistant *S. aureus*) ^a^ MIC = 8 μg/mL (linezolid-resistant *E. faecalis*)	[122]
B/C-ring and C-5 modified			
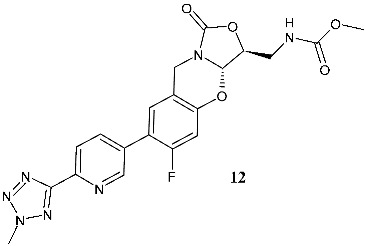	**12** ^a^ MIC = 4 μg/mL (*S. aureus* ATCC25923) ^a^ MIC = 0.5 μg/mL (*E. faecalis* ATCC29212) ^a^ MIC = 0.125 μg/mL (*B. subtilis* ATCC6633) ^a^ MIC = 0.49 μg/mL (*M. tuberculosis* H37Rv) ^a^ MIC = 8–16 μg/mL (linezolid-resistant *S. aureus*) ^a^ MIC = 1–2 μg/mL (linezolid-resistant *E. faecalis*)	Linezolid: ^a^ MIC = 2 μg/mL (*S. aureus* ATCC25923) ^a^ MIC = 0.5 μg/mL (*E. faecalis* ATCC29212) ^a^ MIC = 0.063 μg/mL (*B. subtilis* ATCC6633) ^a^ MIC = 0.8 μg/mL (*M. tuberculosis* H37Rv) ^a^ MIC = 8 μg/mL (linezolid-resistant *S. aureus*) ^a^ MIC = 8 μg/mL (linezolid-resistant *E. faecalis*)	[122]
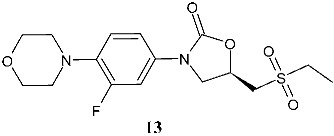	**13** ^a^ MIC = 2.01 μM (*M. tuberculosis* H37Rv) ^a^ MIC = 0.92 μM (MDR *M. tuberculosis*)	Linezolid: ^a^ MIC = 2.31 μM (*M. tuberculosis* H37Rv) ^a^ MIC = 0.81 μM (MDR *M. tuberculosis*)	[86]

^a^ MICs were measured using the standard broth microdilution method according to the guidelines established by the Clinical and Laboratory Standards Institute (CLSI). ^b^ MICs were measured using the agar dilution method according to the guidelines established by the Clinical and Laboratory Standards Institute (CLSI).

## Data Availability

No new data were created or analyzed in this study.

## Supplementary Materials

The following supporting information can be downloaded at: https://www.mdpi.com/article/10.3390/ph19030432/s1, Table S1. Clinical Studies Involving 2-Oxazolidinones.

## Abbreviations

The following abbreviations are used in this manuscript:

*A. baumannii*	*Acinetobacter baumannii*
ABSSSIs	acute bacterial skin and skin structure infections
BDLC	bedaquiline, delamanid, linezolid, and clofazimine
BDLLfxC	bedaquiline, delamanid, linezolid, levofloxacin and clofazimine
BPaL	bedaquiline, pretomanid and linezolid
BPaL(M)	bedaquiline, pretomanid, linezolid and moxifloxacin
BPaLMZ	bedaquiline, pretomanid, linezolid, moxifloxacin, and pyrazinamide
*B. subtilis*	*Bacillus subtilis*
*E. faecalis*	*Enterococcus faecalis*
*E. faecium*	*Enterococcus faecium*
FDA	US Food and Drug Administration
*M. abscessus*	*Mycobacterium abscessus*
MAO	monoamine oxidase
MDR	multidrug-resistant
MDR-TB	multidrug-resistant tuberculosis
*M. smegmatis*	*Mycobacterium smegmatis*
MRSA	methicillin-resistant Staphylococcus aureus
MRSE	methicillin-resistant Staphylococcus epidermidis
MSSE	methicillin-susceptible Staphylococcus epidermidis
*M. tuberculosis*	*Mycobacterium tuberculosis*
*P. aeruginosa*	*Pseudomonas aeruginosa*
PTC	peptidyl transferase center
RR-TB	rifampicin resistant tuberculosis
*S. aureus*	*Staphylococcus aureus*
*S. epidermidis*	*Staphylococcus epidermidis*
SSTIs	skin and soft tissue infections
VRE	vancomycin resistant Enterococcus
WHO	World Health Organization
XDR-TB	extensively drug-resistant tuberculosis
