# Supplementary material for: Current Status and Perspectives of Antibacterial Agents Belonging to 2-Oxazolidinones
Source: Pharmaceuticals (Basel). 2026 Mar 6;19(3):432. doi: 10.3390/ph19030432 (PMC13028916; doi:10.3390/ph19030432)
Supplement: Supplementary file 1 [file pharmaceuticals-19-00432-s001.zip › pharmaceuticals-4173362-supplementary.pdf]

Supporting Information

Current Status and Perspectives of Antibacterial Agents Belonging to 2-Oxazolidinones

Jessica Ceramella, Annaluisa Mariconda, Domenico Iacopetta, Maria Marra, Alessia Catalano*, Paola Checconi, Stefano Aquaro, Carmela Saturnino, Pasquale Longo and Maria Stefania Sinicropi

Table S1. Clinical Studies Involving 2-Oxazolidinones

"Completed" NCT are highlighted in green, "recruiting" in fuscia, "active, not recruiting" in yellow, "enrolling by invitation" in blue, and "not yet recruiting" in gray.

Title of the Clinical Trial	Number	Status	Study type	Phase	Study Completion (actual)	Study Completion (estimated)
**LINEZOLID**						
Two-month Regimens Using Novel Combinations to Augment Treatment Effectiveness for Drug-sensitive Tuberculosis (TRUNCATE-TB)	NCT03474198	Completed	Interventional	III	2022-01-20	
Pharmacokinetic Study of Linezolid for TB Meningitis (SIMPLE)	NCT03537495	Completed	Interventional	II	2023-07-20	
A PAN-USR TB Multi-Center Trial	NCT06905522	Recruiting	Interventional	III		2029-12
Therapeutic Drug Monitoring for Linezolid in the Treatment of Rifampin-resistant Tuberculosis	NCT06590428	Not yet recruiting	Interventional	not applicable		2029-09-01
Triage Test for All Oral DR-TB Regimen (TRiAD Study)	NCT05175794	Completed	Observational	-	2025-06-01	
Linezolid Dosing Strategies in Drug-Resistant TB	NCT05007821	Active, not recruiting	Interventional	II		2026-03-13
Evaluating Newly Approved Drugs for Multidrug-resistant TB	NCT02754765	Completed	Interventional	III	2023-06	
Linezolid for Syphilis Pilot Study	NCT05548426	Recruiting	Interventional	II		2026-03
Alternative Antibiotics for Syphilis	NCT05069974	Active, not recruiting	Interventional	III		2026-04
Comprehensive Ambulatory Antibiotics for the Treatment of Congenital Syphilis (Cares-1)	NCT06921213	Not yet recruiting	Interventional	II		2027-06-30
Model-informed Precision Dosing for Linezolid	NCT06444802	Not yet recruiting	Interventional	III		2026-06-30
Linezolid Plus Standard of Care	NCT06958835	Recruiting	Interventional	not applicable		2028-10
Optimizing Linezolid Dosing in Patients with Advanced Renal Impairment: a Therapeutic Drug Monitoring-based Evaluation	NCT07138521	Not yet recruiting	Interventional	IV		2026-05-15
Adjunctive Linezolid for the Treatment of Tuberculous Meningitis (ALTER)	NCT04021121	Completed	Interventional	II	2023-12-04	
Trial of a Six-Month Regimen of High-Dose Rifampicin, High-Dose Isoniazid, Linezolid, and Pyrazinamide Versus a Standard Nine-Month Regimen for the Treatment of Adults and Adolescents with Tuberculous Meningitis	NCT05383742	Recruiting	Interventional	II		2029-09-15
Intensified Tuberculosis Treatment to Reduce the Mortality of Patients with Tuberculous Meningitis (INTENSE-TBM)	NCT04145258	Recruiting	Interventional	III		2026-04
Linezolid or Vancomycin Surgical Site Infection Prophylaxis	NCT05571722	Recruiting	Interventional	IV		2026-04-03
Linezolid for Treatment of Nontuberculous Mycobacterial Diseases	NCT03220074	Completed	Interventional	IV	2022-06-15	
Finding the Optimal Regimen for Mycobacterium abscessus Treatment (FORMaT)	NCT04310930	Recruiting	Interventional	II/III		2030-06-30
Bacteriophage Therapy for Mycobacterium abscessus Pulmonary Infection	NCT07228702	Enrolling by invitation	Interventional	I		2027-10
Early Intravenous to Oral Antibiotic Switch in Uncomplicated Staphylococcus aureus Bacteraemia	NCT06336824	Recruiting	Interventional	III		2025-06

International Surveillance of Antimicrobial Resistance in Cirrhosis-Related Infections	NCT06634940	Recruiting	Observational	–		2031-06-01
Repurposing Clinically Approved Drugs for Yaws with an Insight into the Cutaneous Ulcer Disease Syndrome (Trep-AByaws)	NCT05764876	Completed	Interventional	III	2024-12-01	
Compare Linezolid-Induced Thrombocytopenia in Patients with Normal Renal Function Versus Impaired Renal Function (LinezRenal)	NCT06761131	Completed	Observational	–	2025-06-20	
Linezolid, Aspirin and Enhanced Dose Rifampicin in HIV-TBM	NCT03927313	Completed	Interventional	II	2021-03-31	
Efficacy of Intravenous N-Acetylcysteine in Preventing Linezolid-Induced Thrombocytopenia in Critically Ill Patients	NCT05944458	Completed	Interventional	IV	2025-06-01	
In Vivo Antibiotics Removal During Hemoadsorption Cartridges and Continuous Renal Replacement Therapy in the Intensive Care Unit	NCT07230041	Recruiting	Observational	–		2026-02-15
Population Pharmacokinetics of Commonly Used Antimicrobial Agents in Children of Bacterial Meningitis with Augmented Renal Clearance	NCT04771884	Recruiting	Observational	–		2026-03-20
Studying the Distribution of Accessory Gene Regulator (Agr) Quorum Sensing System and the Prevalence of Linezolid and Mupirocin Resistance in Biofilm Producer/Non Producer Staphylococcus aureus in Sohag University Hospitals	NCT06291181	Completed	Interventional	not applicable	2023-06-30	
NextGen - Clinical Implication of Next Generation Sequencing	NCT05206500	Recruiting	Interventional	IV		2028-12
FOUR-SIX TRIAL for the Treatment of Enterococcal Endocarditis (FOURSIX)	NCT05398289	Not yet recruiting	Interventional	IV		2026-09-01
Impact of Rapid Pathogen Detection in ICU Patients with Suspected Pneumonia on Antimicrobial Therapy (IRISPAT-1)	NCT06478953	Recruiting	Interventional	II		2025-12-31
Clinical Trial Testing Whether Targeted Antibiotic Prophylaxis Can Reduce Infections After Cystectomy Compared to Empiric Prophylaxis (REINFORCE)	NCT06709196	Recruiting	Interventional	IV		2027-01
Postoperative Antibiotic Management Duration Following Surgery for Intravenous Drug Abuse (IVDA) Endocarditis (OPTIMAL)	NCT05156437	Enrolling by invitation	Interventional	IV		2024-10
Rapid De-escalation of Anti-MRSA Therapy Guided by S. aureus Nares Screening in Case of Pneumonia (SNAP)	NCT06238297	Recruiting	Interventional	not applicable		2025-06-30
Concentration Monitoring of Anti-infective Drugs in Human Cerebrospinal Fluid and Its Clinical Application	NCT06729619	Active, not recruiting	Observational	–		2027-12-31
Efficacy of Empirical Anti-Infective Therapy in Neutropenic Febrile Patients	NCT07204522	Recruiting	Observational	–		2027-09-22

TEDIZOLID

Evaluation of the Early Bactericidal Activity of Tedizolid and Linezolid Against Mycobacterium Tuberculosis (TEDITUB)	NCT05534750	Recruiting	Interventional	II		2026-05
Tedizolid Suppressive Antimicrobial Therapy in a Reference Center (TediSAT)	NCT04662736	Completed	Observational	–	2021-04-01	
Tedizolid Neuropathies	NCT07143240	Completed	Observational	–	2025-05-01	
Tolerability, Safety, and Efficacy of Tedizolid as Oral Treatment for Bone and Joint Infections (OTTER)	NCT03009045	Completed	Interventional	II	2021-08-06	
Tedizolid Prolonged Treatment for Prosthetic Joint Infections (TEDIZOAM)	NCT03378427	Completed	Interventional	not applicable	2021-08-22	

Efficacy and Tolerance of 4 Weeks of Tedizolid in Prosthetic Joint Infections Treated with Implant Removal (PROTEDI)	NCT03746327	Completed	Interventional	IV	2024-03-10	
A Study to Evaluate Oral Formulations of Tedizolid Phosphate in Healthy Participants (MK-1986-043)	NCT06733688	Completed	Interventional	I	2022-04-15	
A Pharmacokinetic Study of Tedizolid Phosphate in Pediatric Participants with Gram-Positive Infections (MK-1986-014)	NCT03217565	Completed	Interventional	I	2023-04-06	
A Study to Compare Oral Formulations of Tedizolid Phosphate in Healthy Adults (MK-1986-044)	NCT06609161	Completed	Interventional	I	2024-08-08	
A Study of Safety and Efficacy of MK-1986 (Tedizolid Phosphate) and Comparator in Participants from Birth to Less Than 12 Years of Age with Acute Bacterial Skin and Skin Structure Infections (MK-1986-018)	NCT03176134	Completed	Interventional	III	2023-07-06	
Pathogenicity Factors of Staphylococcus Pettenkoferi in Foot Wounds and Osteitis in Diabetic Patients (PETTENK-OS)	NCT06688084	Active, not recruiting	Observational	–		2026-05-01
Oral Antimicrobial Treatment vs. Outpatient Parenteral for Infective Endocarditis (OraPAT-IEGAMES)	NCT05398679	Recruiting	Interventional	IV		2027-12-30

CONTEZOLID

Phase II Clinical Study of Contezolid for the Treatment of Tuberculous Meningitis	NCT06811025	Enrolling by invitation	Interventional	II		2025-12-31
Short-term Bactericidal Effect of Contezolid in MAC-PD (CONTE-MAC)	NCT07084402	Recruiting	Interventional	IV		2027-12-31
Phase II Clinical Study of Contezolid for the Treatment of Bone and Joint Tuberculosis	NCT06811012	Enrolling by invitation	Interventional	I, II		2025-12-31
Safety and Efficacy Study of Contezolid Acefosamil and Contezolid Compared to Linezolid Administered Intravenously and Orally to Adults with Moderate or Severe Diabetic Foot Infections (DFI)	NCT05369052	Recruiting	Interventional	III		2026-06-30
Short-term Antibiotic Therapy in Mycobacterium avium Complex Pulmonary Disease	NCT07213765	Recruiting	Interventional	IV		2028-12-31
Contezolid Acefosamil Versus Linezolid for the Treatment of Acute Bacterial Skin and Skin Structure Infection	NCT03747497	Completed	Interventional	II	2019-03-26	

RADEZOLID

Safety and Efficacy Study of Oxazolidinone to Treat Pneumonia	NCT00640926	Completed	Interventional	II	2009-04	
Safety and Efficacy Study of Oxazolidinones to Treat Uncomplicated Skin Infections	NCT00646958	Completed	Interventional	II	2008-04	

SUTEZOLID (PNU-100480)

Trial of Novel Regimens for the Treatment of Pulmonary Tuberculosis (RAD-TB)	NCT06192160	Recruiting	Interventional	II		2027-08-11

PanACEA Sutezolid Dose-finding and Combination Evaluation (SUDOCU)	NCT03959566	Completed	Interventional	II	2022-09-30	
Study to Evaluate the Safety, Tolerability, and Pharmacokinetics of Sutezolid	NCT03199313	Completed	Interventional	I	2017-12-16	
Safety, Tolerability and Pharmacokinetics Study of Single Doses Of PNU-100480 In Healthy Adults	NCT00871949	Completed	Interventional	I	2009-08	
PNU-100480 In Newly Diagnosed, Drug Sensitive Patients with Pulmonary TB; Early Bactericidal Activity; Whole Blood Assay	NCT01225640	Completed	Interventional	II	2011-12	
Safety, Tolerability, Pharmacokinetics and Measurement of Whole Blood Activity (WBA) Of PNU-100480 After Multiple Oral Doses in Healthy Adult Volunteers	NCT00990990	Completed	Interventional	I	2010-05	
Efficacy and Safety Evaluation of Two to Four Months of Treatment with the Combination Regimens of DBOS and PBOS in Adults with Pulmonary Tuberculosis	NCT05971602	Terminated	Interventional	II	2025-02-06	
The Individualized M(X) Drug-resistant TB Treatment Strategy Study (InDEX)	NCT03237182	Terminated	Interventional	IV	2022-12-19	

DELPAZOLID (LCB01-0371)

Trial to Evaluate Bioequivalence of LCB01-0371 in Different Batches	NCT04939779	Completed	Interventional	I	2021-02-01	
A Phase 2a Study, Effect of Vancomycin With vs Without Delpazolid (LCB01-0371) in Patients with MRSA Bacteremia	NCT05225558	Terminated	Interventional	II	2024-03-18	
Study to Evaluate the Efficacy of Delpazolid as Add-on Therapy in Refractory *Mycobacterium abscessus* Complex	NCT06004037	Active, not recruiting	Interventional	II		2026-12-30
PanACEA - STEP2C-01	NCT05807399	Recruiting	Interventional	II		2027-12-30
Platform Assessing Regimens and Durations in a Global Multisite Consortium for TB	NCT06114628	Recruiting	Interventional	II		2027-08-11

TBI-223

Study to Evaluate Safety, Tolerability, and the PK Profile of TBI-223 in Healthy Subjects	NCT04865536	Completed	Interventional	I	2022-05-17	
A Phase 1 Study to Evaluate Safety, Tolerability, and Pharmacokinetics of TBI-223 in Healthy Adults	NCT03758612	Completed	Interventional	I	2020-03-15	

MK-7762 (TBD09)

A Study to Evaluate the Safety, Tolerability, and Pharmacokinetics of TBD09	NCT07094932	Active, not recruiting	Interventional	I		2025-12-13
Safety, Tolerability, Pharmacokinetics (PK), and Food Effect of MK-7762 in Healthy Adults	NCT05824091	Completed	Interventional	I	2024-03-26	

BPaL, BPAL(M)

A Phase 3 Trial Assessing Safety and Efficacy of B-Pa-L in Participants With DR-TB	NCT02333799	Completed	Interventional	III	2020-08-03	

6 Months of Bedaquiline(BDQ), Delamanid(DLM), Linezolid(LZD) and Levofloxacin(LFX) in RR-TB Patients in Hubei Province (BDLL)	NCT07198685	Not yet recruiting	Interventional	not applicable		2028-09-30
The Safety and Efficacy of BDL(Bedaquiline Plus Delamanid Plus Linezolid) Regimen in Subjects With Pulmonary Infection of Multi-drug Resistant Tuberculosis (MDR-TB) or Rifampicin-Resistant Tuberculosis (RR-TB)	NCT06476210	Recruiting	Interventional	IV		2026-06
BPaL(M) Regimen for the Treatment of MDR/RR-TB	NCT05381194	Recruiting	Interventional	IV		2027-12
Efficacy and Tolerability of Bedaquiline, Delamanid, Levofloxacin, Linezolid, and Clofazimine to Treat MDR-TB (DRAMATIC)	NCT03828201	Recruiting	Interventional	II		2027-05-31
Building Evidence for Advancing New Treatment for Rifampicin Resistant Tuberculosis (RR-TB) Comparing a Short Course of Treatment (Containing Bedaquiline, Delamanid and Linezolid) With the Current South African Standard of Care	NCT04062201	Completed	Interventional	III	2024-04-15	
Modified BPaL Regimen for Managing Pre-XDR TB and MDR (TI/NR) TB in India (mBPaL)	NCT05040126	Active, not recruiting	Interventional	III		2024-12-31
Phase 2 Trial Assessing TBAJ876 or Bedaquiline, with Pretomanid and Linezolid in Adults with Drug-sensitive Pulmonary Tuberculosis	NCT06058299	Active, not recruiting	Interventional	II		2026-06
Various Doses and Durations of Linezolid Plus Bedaquiline & Pretomanid in Participants with Drug Resistant Tuberculosis (ZeNix)	NCT03086486	Completed	Interventional	III	2022-02-08	
PanACEA DElpazolid Dose-finding and COmbination DEvelopment (DECODE)	NCT04550832	Completed	Interventional	II	2023-09-11	
Program for Rifampicin-Resistant Disease with Stratified Medicine for Tuberculosis (PRISM-TB)	NCT06441006	Recruiting	Interventional	II/III		2031-08
B-PaLMZ for TB Meningitis	NCT07227779	Not yet recruiting	Interventional	II		2030-08-31

Other Combination Therapies

Innovating Shorter, All-Oral, Precised, Individualized Treatment Regimen for Rifampicin Resistant Tuberculosis: Contezolid, Delamanid and Bedaquiline Cohort (INSPIRE-CODA)	NCT06081361	Active, not recruiting	Interventional	III		2026-12-31
Refining MDR-TB Treatment (T) Regimens (R) for Ultra(U) Short(S) Therapy(T)-PLUS (TB-TRUSTplus)	NCT04717908	Completed	Interventional	not applicable	2024-06-22	
An Open-label RCT to Evaluate a New Treatment Regimen for Patients with Multi-drug Resistant Tuberculosis (NEXT)	NCT02454205	Completed	Interventional	II/III	2021-08-30	
Platform Assessing Regimens and Durations In a Global Multisite Consortium for TB (PARADIGM4TB)	NCT06114628	Recruiting	Interventional	II		2027-08-11
Innovating(IN) Shorter(S), All- Oral, Precised(P), Individualized(I) Treatment Regimen(RE) for Rifampicin Resistant Tuberculosis(INSPIRE-TB)	NCT05081401	Recruiting	Interventional	III		2027-12-01
Evaluating Newly Approved Drugs in Combination Regimens for Multidrug-Resistant TB with Fluoroquinolone Resistance (endTB-Q)	NCT03896685	Completed	Interventional	III	2024-12-31	
Innovating Shorter, All- Oral, Precised Treatment Regimen for Rifampicin Resistant Tuberculosis: BLMZ Chinese Cohort (INSPIRE-BLMZ)	NCT07268664	Not yet recruiting	Interventional	III		2028-08-31

Patient-reported Experiences and Quality of Life Outcomes in the TB-PRACTECAL Clinical Trial (PRACTECAL-PRO)	NCT03942354	Completed	Observational	–	2022-05-10	
Pragmatic Clinical Trial for a More Effective Concise and Less Toxic MDR-TB Treatment Regimen(s) (TB-PRACTECAL)	NCT02589782	Completed	Interventional	II/III	2022-08-05	
Registry on the Effectiveness and Safety of the 9-month MDR-END Treatment Regimen in Korean Patients with FQ-sensitive MDR-TB (MDR-END Registry)	NCT06674291	Not yet recruiting	Observational	–		2028-12-31
Molecular Epidemiology, Strains Genotyping of Multidrug Resistant Tuberculosis Circulating in Central Africa Region (MESTCAR)	NCT07017803	Not yet recruiting	Observational	–		2026-01-31
A Study on the Short - Course Treatment Regimen Containing Pretomanid for Diabetes Mellitus Complicated with Rifampicin-Resistant/Multidrug-Resistant Pulmonary Tuberculosis	NCT07191834	Not yet recruiting	Observational	–		2027-09-30
Reducing Antimicrobial Overuse Through Targeted Therapy for Patients with Community-Acquired Pneumonia	NCT05568654	Recruiting	Interventional	not applicable		2026-06-30
